# Comparison of Dietary Micronutrient Intakes by Body Weight Status among Mexican-American and Non-Hispanic Black Women Aged 19–39 Years: An Analysis of NHANES 2003–2014

**DOI:** 10.3390/nu11122846

**Published:** 2019-11-20

**Authors:** Jialiang Liu, Xiangzhu Zhu, Kimberly G. Fulda, Shande Chen, Meng-Hua Tao

**Affiliations:** 1 Department of Biostatistics and Epidemiology, University of North Texas Health Science Center, Fort Worth, TX 76107, USA; Jialiang.Liu@live.unthsc.edu; 2 Department of Medicine, Division of Epidemiology, Vanderbilt Epidemiology Center, Vanderbilt University School of Medicine, Vanderbilt-Ingram Cancer Center, Vanderbilt University Medical Center, Nashville, TN 37203, USA; xiangzhu.zhu@Vanderbilt.Edu; 3 Department of Family Medicine and Osteopathic Manipulative Medicine, NorTex, University of North Texas Health Science Center, Fort Worth, TX 76107, USA; kimberly.fulda@unthsc.edu; 4 Graduate School of Biomedical Sciences, University of North Texas Health Science Center, Fort Worth, TX 76107, USA; Shande.Chen@unthsc.edu

**Keywords:** micronutrient, overweight, obesity, Mexican-American, non-Hispanic Black, women

## Abstract

The objective of the current study was to examine micronutrient intake from foods in women of childbearing age and to better understand potential nutritional problems varied by body weight status in minority women. A sample of women aged 19–39 years from the National Health and Nutrition Examination Surveys (NHANES) 2003–2014 was analyzed. Dietary intakes of 13 micronutrients were estimated using the National Cancer Institute method. Mexican-American and non-Hispanic Black women were categorized into normal/under-weight, overweight, or obese groups according to their body mass index (BMI). Mexican-American and non-Hispanic Black women had lower dietary intakes for vitamins A, B_2_, B_6_, B_12_, and D, folate, calcium, and magnesium than non-Hispanic Whites. Among Mexican-Americans, obese women had the lowest dietary intake of vitamins A, B_2_, C and D. Obese non-Hispanic Black women had significantly lower dietary intakes of iron and zinc than their normal/under-weight counterparts. Comparable percentages (>30%) of Mexican-American and non-Hispanic Black women had dietary intake less than the Estimated Average Requirements (EARs) for several key nutrients including vitamin A, C and D, folate, calcium and magnesium, and the percentages varied by body weight status. These results indicate micronutrient inadequacies persist among and within racial/ethnic and body weight groups.

## 1. Introduction

For women of childbearing age, good diet is essential to achieve a healthy pregnancy, lactation and birth outcomes [1,2], and to maintain health conditions in later life for both mother and child [2,3,4,5,6]. Previous studies have shown associations of insufficient micronutrient intakes during the pre- and post-conception period with adverse pregnancy outcome, increased risk of maternal complications during the pregnancy, and chronic diseases in later life [7,8,9,10]. Low vitamin A level in the diet during pregnancy was associated with increased risk of fetuses having congenital diaphragmatic hernia, or schizophrenia spectrum disorders in children [7]. A cohort study conducted in South Korea reported that higher maternal vitamin C intake was associated with greater infant birth length and reduced risk of having a lower birth weight [8]. Another prospective cohort study found that Chinese women of childbearing age with high dietary vitamin C intake during pregnancy had lower risk of gestational diabetes [9], and women with high intake of magnesium during pregnancy were positively associated with bone mineral density in their offspring at age 16 years [10].

Nutritional intake is associated with the risk of weight gain and obesity for women of childbearing age [11], but also is highly dependent on ethnic/cultural environment and personal dietary habits [3]. A number of studies have shown correlations between low dietary intake of micronutrients, including vitamins A, C, D and E, calcium, magnesium and zinc, and obesity and body fat mass [12,13,14]. In 2015–2016, 36.5% of American women of childbearing age (20–39 years) were classified as obese [15], and the prevalence was particularly high in non-Hispanic Black and Hispanic women compared to non-Hispanic White women [15,16]. Previous research on nutrient intake among American women of childbearing age primarily focused on differences in intakes by race/ethnicity and age categories [17,18,19]. Previous results showed lower intakes for fiber, folate, riboflavin, phosphorus, potassium, calcium and magnesium among Black women of childbearing age compared to White and Mexican-American counterparts [16], and a significant percentage of women not meeting the Estimated Average Requirement (EAR) for several nutrients such as vitamins A and D, and fiber regardless of their race/ethnicity [17].

To date, we have little understanding of micronutrient status or inadequacies by body composition status among U.S. women of childbearing age, particularly in racial/ethnic minority populations [20]. We focused on micronutrient status because several essential micronutrients such as vitamins A, C and D, calcium, and magnesium have been recognized as nutrients of concern for the general adult population according to the Dietary Guidelines for Americans 2015–2020 [21], and intakes of iron, folate and zinc are of critical concern, particularly among women of childbearing age due to high requirements and losses [5,21]. To help address this gap and contribute to develop targeted dietary supplement programs for specific subgroups in racial/ethnic populations, usual daily micronutrient intakes from foods among women aged 19–39 years from the National Health and Nutrition Examination Survey (NHANES), 2003–2014, were analyzed by race/ethnicity and body mass index (BMI). The objectives of this study were to compare whether usual daily micronutrient intakes from foods vary by BMI status among Mexican-American and non-Hispanic Black women of childbearing age, and to determine the prevalence of nutritional inadequacies by BMI status in each racial/ethnic subpopulation.

## 2. Materials and Methods

### 2.1. Study Population and Anthropometry Data

The current study utilized data from the continuous NHANES survey, encompassing from 2003 through to 2014, six NHANES survey cycles. The NHANES is a cross-sectional survey designed to monitor the health and nutritional status of a nationally representative sample of the noninstitutionalized civilian U.S. population [22]. The NHANES data are released every 2 years by the National Center for Health Statistics (NCHS) of the Centers for Disease Control and Prevention in the USA [22]. Our study population included women, aged 19–39 years at the time of the survey, and self-identified race/ethnicity as non-Hispanic White, Mexican-American or non-Hispanic Black (*N* = 5406). Women who self-identified as other Hispanic were not included in the current analysis due to changes in sampling structure of the Hispanic population over NHANES cycles. Pregnant or lactating women, as well as those with missing data for dietary micronutrient intakes, height, or weight were further excluded from the analysis. Finally, there were 1916 White, 916 Mexican-American and 1099 non-Hispanic black women between 19 and 39 years of age included in the analyses. All participants provided written informed consent, and the Research Ethics Review Board at the National Center for Health Statistics (NCHS) approved the survey protocol [22]. 

Height and weight were measured in NHANES mobile examination centers (MEC) according to validated anthropometry manual standards [23]. Body mass index (BMI) was calculated as body weight in kilograms divided by the square of height in meters. For the current analyses, the authors focused on comparisons across body weight groups in Mexican-American and non-Hispanic Black women aged 19–39 years. Among women aged 19–39 years, only 1.6% (15) of Mexican Americans and 3.0% (36) of non-Hispanic Blacks were classified as underweight according to World Health Organization criteria [24], which limited statistical ability to estimate dietary intake of micronutrients for those women. Finally, the authors classified women from these two racial/ethnic populations into three groups: <25.0, 25.0–29.9, and ≥30.0 for groups of normal/under-weight, overweight and obese, respectively.

### 2.2. Assessments of Micronutrient Intakes from Foods

Daily dietary intake information including types and amounts of individual foods and components of food preparation methods such as use of fat and salt in preparation, cooking methods and type of liquid added in recipe foods were collected through two 24-hour dietary recall interviews using the U.S. Department of Agriculture’s Automated Multiple-Pass method. The first dietary recall (Day 1) was collected in person by trained interviewers in the MEC. The second dietary recall (Day 2) was completed by telephone approximately 3–10 days after the MEC interview [25]. The quantitative micronutrients intakes were calculated by using various U.S. Department of Agriculture’s (USDA) food composition databases, which was described in detail elsewhere [26]. The USDA databases estimated the nutrient content of NHANES foods in recipes by linking the ingredients in the survey food recipes to food composition data [25]. Only dietary recall data verified as reliable by trained study staff were used in the analyses.

### 2.3. Statistical Analysis

Mean daily dietary intake and standard error (SE) for 13 micronutrients and total energy were estimated by race/ethnicity, and by BMI status in each racial/ethnic subpopulation. EAR represents the average daily nutrient intake level that meets the adequacy requirement for half of the healthy individuals in a life stage or gender group [27], and the U.S. Institute of Medicine (IOM) released the most recent EARs as recommendations for most usual micronutrient intakes in 2006 [27]. The prevalence of women not meeting the EAR were further estimated for selected micronutrients in each racial/ethnic subpopulation separately by their BMI status. 

Considering the day-to-day variation (also referred to as within-person variation) in individual diets and the complex sample design of NHANES, the National Cancer Institute (NCI) method with the balanced repeated replication (BRR) weights was used in the current study to estimate usual dietary micronutrient intakes and the percentage of women not meeting the EARs in different subgroups [28]. Two 24-hour dietary recalls (day 1 or day 2) were used while applying the NCI method. As previously described [28,29,30], the NCI method uses a mixed-effects model, which is the extension of simple linear regression models to allow both random and fixed effects to separate the within-person from between-person variation to obtain an estimated distribution that reflects variation in the usual intake of micronutrients. The NCI model separates usual intake into two parts: the probability to consume a food on a particular day, and given that the food was consumed, the amount eaten on the consumption day [29]. In the present study, because all micronutrients were considered to be ubiquitously consumed by a majority of the population, the probability of consuming a micronutrient on a given day was close to or equal to 1, only the amount part of the NCI model was used to estimate the usual intakes of each micronutrient [28,30]. The NCI model included covariates of the period of a week of dietary recall (i.e., weekend/ weekday), and the sequence of 24-hour recall (i.e., day 1 or day 2) to account for the day-to-day variation of intake in the usual intake estimation process. Because the requirement distribution for iron is asymmetrical for women [31], the full probability method was used to estimate the prevalence of individuals with iron intake below the EAR. Linear regression models were used to evaluate whether the mean usual dietary intake of micronutrient differs by race/ethnicity in the whole study population and whether the usual intake differs by BMI status in every individual racial/ethnic subpopulation through the incorporation of covariates (i.e., race/ethnicity, BMI status) into the models. All statistical analyses were performed with Statistical Analysis System (SAS) 9.4 version [32]. Statistical tests were two tailed, with an overall significance level of 0.05.

## 3. Results

Table 1 shows the selected characteristics of the study population and their BMI status. As expected, non-Hispanic Black women had the highest prevalence of overweight and obesity (74.5%), followed by Mexican-American women (69.8%), and non-Hispanic White women (51.4%). Non-Hispanic Whites had higher poverty–income ratio (PIR) and education attainment than non-Hispanic Blacks and Mexican-Americans, and non-Hispanic Blacks were least likely to be married. 

The usual intakes of micronutrients from foods by race/ethnicity are shown in Table 2. Among 19–39-year-old women, the intakes of the following micronutrients were significantly different across the three racial/ethnic groups: vitamins A, B_2_, B_6_, B_12_, C, D, folate, calcium, magnesium, and copper (*p* ≤ 0.05). Mexican-American women had the lowest dietary intakes of these micronutrients, except that non-Hispanic White women had the lowest intake of vitamin C (*p* ≤ 0.05). There was no significant difference observed in usual dietary intakes across racial/ethnic groups for phosphorus, copper, iron, and zinc (*p* > 0.05). 

The usual dietary intakes of micronutrients were further estimated by BMI status among Mexican-American and non-Hispanic Black women (Table 3 and Table 4). Among Mexican-American women, usual dietary intakes for vitamins A, B_2_, C, D, and phosphorus were significantly different by their BMI status (*p* ≤ 0.05) (Table 3). Compared to normal/under-weight women, the lowest dietary intakes of vitamins A, B_2_, and C were found in obese Mexican-American women, followed by the overweight group. Obese women also had significantly lower dietary intake of vitamin D, while overweight women had significantly higher intake of phosphorus than normal/under-weight women (*p* ≤ 0.05). The usual dietary intakes for vitamins B_6_, B_12_, folate, calcium, magnesium, copper, iron, zinc, and energy were not significantly different by BMI status among Mexican-American women.

In non-Hispanic Black women, there were no significant differences found in micronutrient intakes from foods by BMI status for a majority of micronutrients such as vitamins A, B_2_, B_6_, B_12_, C, and D, folate, calcium, magnesium, phosphorus, and copper except for iron and zinc. Dietary intake for iron and zinc were significantly different across BMI groups in non-Hispanic Black women (*p* ≤ 0.05), with the lowest intake for these two micronutrients in the obese group (Table 4). 

**Figure 1:**
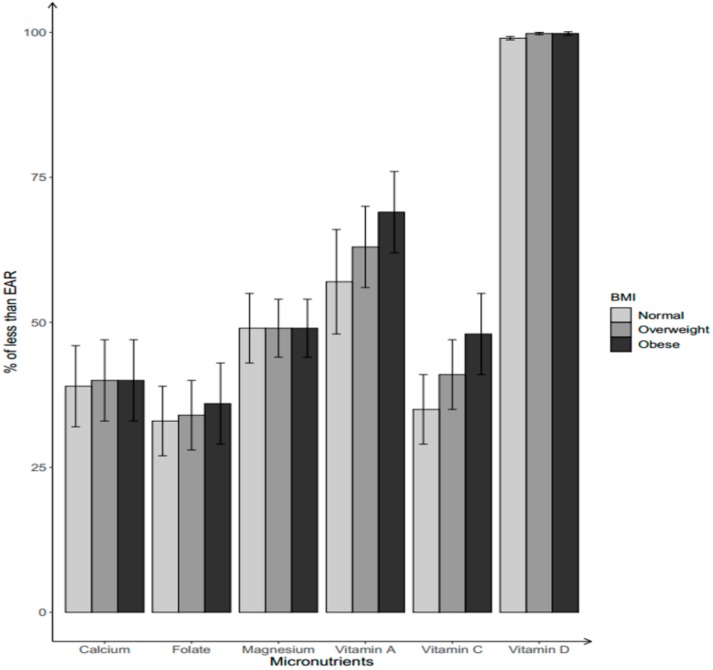
Percentage of Mexican-American women aged 19–39 years with dietary micronutrient intakes below the Estimated Average Requirements (EARs) by body weight status. Body weight status categorized by BMI into three groups: normal/under-weight: BMI ≤ 25.0; overweight: 25.0 < BMI < 29.9; obese: BMI ≥ 30.0.

**Figure 2:**
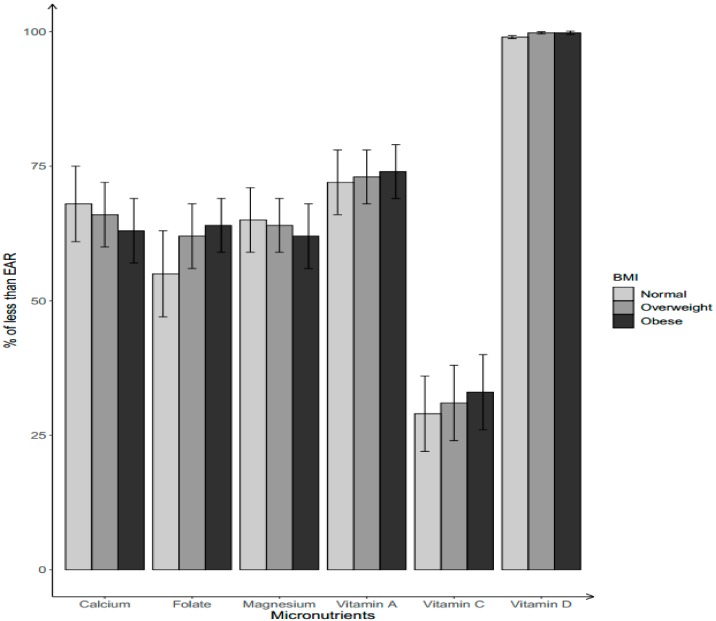
Percentage of non-Hispanic Black women aged 19–39 years with dietary micronutrient intakes below EARs by body weight status. Body weight status categorized by BMI into three groups: normal/under-weight: BMI ≤ 25.0; overweight: 25.0 < BMI < 29.9; obese: BMI ≥ 30.0.

The percentages of women aged 19–39 years having dietary intakes of micronutrients below the EARs, for which more than 30% of the population failed to meet the EARs, are presented in Figure 1 for Mexican-Americans and Figure 2 for non-Hispanic Blacks. Figure 1 shows that 57%–69% of Mexican-American women did not meet the EAR for vitamin A, with the noticeably highest prevalence for the obese group. Likewise, 48% of obese Mexican-American women did not meet the EAR for vitamin C intake from foods, greater than other BMI groups. For dietary vitamin D intake, close to 100% of Mexican-American women failed to meet the EAR regardless of their BMI status. Meanwhile, 33%–37% of Mexican-American women were not meeting the EARs for dietary intake of folate. The prevalence of not meeting EAR was 40% for dietary calcium in both overweight and obese groups, and 49% of Mexican-American women in all BMI groups consumed magnesium intake from foods less than the EAR.

Among non-Hispanic Black women, the percentage of having a usual intake below the EAR tended to be higher in the normal/under-weight group for calcium (68.0%) and magnesium (65.0%), followed by overweight (calcium: 66.0%; magnesium: 64.0%) and obese women (calcium: 63.0%; magnesium: 62.0%) (Figure 2). Obese non-Hispanic Black women have the highest prevalence of not meeting the EAR for vitamins A (74.0%), and C (33.0%), and folate (64.0%), followed by overweight (vitamin A: 73.0%; vitamin C: 31.0%; folate: 62.0%) and normal/under-weight women (vitamin A: 72.0%; vitamin C: 29.0%; folate: 55.0%). Most (99%) of non-Hispanic Black women had a dietary intake below the EAR for vitamin D regardless of their BMI groups.

Similarly, the usual dietary intakes of micronutrients and micronutrient adequacies were estimated by BMI status among non-Hispanic White women aged 19–39 years (Table S1 and Figure S1). Usual dietary intakes for vitamins A, B_2_, B_6_, and C, folate, magnesium, copper, and iron were significantly different by BMI status (*p* ≤ 0.05), with the lowest intakes for those micronutrients in the obese group. Obese women had the highest prevalence of in adequacy for vitamins A and C, folate, calcium and magnesium, followed by overweight and normal/under-weight women. Close to 100% of non-Hispanic White women had dietary vitamin D intake below the EAR in all BMI groups. 

## 4. Discussion

Current study of NHANES 2003–2014 cycles of women aged 19–39 indicated that there were racial/ethnic disparities in dietary intakes for several key micronutrients including vitamins A, B_2_, B_6_, B_12_, C and D, folate, calcium, and magnesium. Except that non-Hispanic White women had the lowest dietary intake of vitamin C, Mexican-American and non-Hispanic Black women tended to have poorer dietary intakes for these micronutrients than non-Hispanic White women. The observed racial/ethnic variations in intakes of micronutrients from foods among U.S. women of childbearing age are consistent with previous reports [17,18,19]. 

There is evidence that obesity is associated with chronic inadequate intakes of micronutrients [12,13,14]; however, the status of micronutrient intake from foods by BMI status has been less examined in minority women of childbearing age [20,34,35,36]. The current study further examined and compared dietary micronutrient intake and the prevalence of women not meeting the EARs for micronutrients by BMI groups in racial/ethnic minority women. Overall, dietary intakes of vitamins A, B_2_, and C were significantly lower in overweight and obese Mexican-American women, which was consistent with a previous study in Mexican women of childbearing age [36]. Consistent with other studies of non-Hispanic Black women [37], the current study found that overweight and obese non-Hispanic Black groups had lower intake of iron and zinc from foods than their normal-weight counterparts. Meanwhile, a large proportion of women did not meet the EARs for several micronutrients including vitamins A, C and D, folate, calcium, and magnesium in each racial/ethnic group, and the prevalence of insufficiencies for vitamins A and C, and folate were particularly high among obese women from the two minority groups.

Vitamin A is essential for vision development, reproduction and embryonic growth [38,39], but also is involved in the regulation of adiposity [40]. An insufficient vitamin A level can pose a significant risk to women of childbearing age, such as anemia and infant growth retardation, that are associated with maternal and infant morbidity and mortality [7]. Consistent with a previous study [17], the current study observed more than 70% and 55% of inadequacies for dietary vitamin A intake for non-Hispanic Black women and Mexican-American women of childbearing age, with the highest prevalence among obese women in the two minority groups.

As an antioxidant nutrient, vitamin C plays a role in defense against obesity-induced oxidative stress through scavenging free radicals and inhibiting lipid peroxidations [13,36]. Insufficient vitamin C intake during pregnancy was also associated with increased risk of low birth weight and weight growth from birth to 6 months for children [8], and a higher risk of gestational diabetes mellitus for the mother [9]. Consistent with prior reports on women of childbearing age [17,41], this study of NHANES 2003–2014 data showed a considerable percentage of women with dietary intake of vitamin C below the EAR among overweight and obese women in all racial/ethnic groups. The particular BMI differentials in dietary vitamins A and C intake for young minority women may suggest the joint contribution of multiple interacting factors such as adiposity distribution and biological influence on nutrient metabolism [42], and indicate that dietary supplementations of vitamins A and C are particularly necessary for young overweight/obese women in these minority populations. 

Folate plays a critical role in DNA synthesis, neurologic functions, and normal red blood cell production [43]. Insufficient folate intake can increase the risk of having a premature or low-birth-weight baby, or neural tube defects (NTD) [44]. Since 1998, folic acid fortification of all enriched cereal-grain products has been applied in the U.S. to reduce the risk of pregnancies affected by NTD among women of childbearing age [44]. However, previous results showed only 10% of women of childbearing age reached the recommended erythrocytes folate level that has been associated with reduced risk of NTDs after fortification [45]. Another study using NHANES 2003–2006 data showed that approximately 83% of Mexican-American and 90% of African-American women aged 15–44 years did not consume the recommended intake of folate [46]. Consistently, the current study found that over 30% and 55% of Mexican-American and non-Hispanic Black women aged 19–39 years failed to meet EAR for folate intake from food sources, and the prevalence were particularly high in overweight and obese non-Hispanic Black women (>60%), suggesting that they may need to take extra folic acid supplements to reach the recommended levels for NTDs prevention. 

One strength of the present study is the use of NHANES data with a large population-based cross-sectional study with a nationally representative sample. Anthropometric measurements were collected by trained research staff at MEC. Dietary intake data from six continuous cycles were combined to provide statistically reliable estimates for the age-, sex-, and race/ethnicity-specific subpopulation of interests. The NCI method was used to estimate the usual micronutrient intake distributions, which represents the gold standard for addressing the statistical challenges when assessing usual nutrient intake among populations [28]. However, there are several limitations to this study. The 24-hour dietary recall used in NHANES has been extensively tested; however, the self-reported dietary recall is likely to have both random and systematic errors, particularly in energy intake [47]. Recall bias from self-reported diet may also exist [48]. Although dietary micronutrient intakes were assessed based on a two-day 24-hour dietary recall, these data may still be insufficient to capture an individual’s usual dietary intake as compared with data obtained from multiple days of food records. Nevertheless, two-day 24-hour dietary recall data have been shown to be an adequate measure for describing dietary intakes at the population level [28]. The current study combined data from eight NANES cycles due to the limited number of eligible women in each NHANES wave. However, there have been significant increases in BMI and dietary intake of many nutrients over the study period from 2003/2004 to 2013/14, which may have influenced our results. Results of the current study provided an important assessment of micronutrient intakes from foods among the U.S. minority population of women of childbearing age, as well as by different BMI status. 

## 5. Conclusions

In conclusion, Mexican-American and non-Hispanic Black women aged 19–39 years across different BMI groups had a poor-quality diet, with intakes well below the EARs for several key micronutrients, and these insufficiencies were more prevalent in overweight/obese groups. Understanding and controlling micronutrient inadequacies for women of childbearing age can have significant public health impact on the health of women of childbearing age, as well as on the health of their offspring. Results from the present study summarized information on key micronutrient concerns for women of childbearing age from target racial/ethnic groups, and provided public health professionals perspectives for developing targeted and/or precise dietary supplement programs or interventions to improve the nutritional status for women of childbearing age from different racial/ethnic groups in the USA.

## Figures and Tables

**Table 1:** Demographic information among women aged 19–39 years by racial/ethnic groups, NHANES 2003–2014 * (*N* = 3931).

	Non-Hispanic White (*n* = 1916)	Mexican-American (*n* = 916)	Non-Hispanic Black (*n* = 1099)	*p*-Value
Age (years)	29.3 ± 0.2	29.1 ± 0.2	28.7 ± 0.3	0.1658
BMI group (%)				<0.0001
Normal/underweight	48.6	30.0	25.6	
Overweight	23.3	30.5	23.1	
Obesity	28.1	39.5	51.2	
Marital status (%)				<0.0001
Married	46.3	47.1	20.4	
Not married	53.7	52.9	79.6	
PIR ^a^ (%)				<0.0001
<1.0	16.6	38.4	35.9	
1.0–1.84	16.9	28.3	24.6	
≥1.85	66.5	33.3	39.5	
Education (%)				<0.0001
Less than high school	8.8	41.9	17.2	
High school/ GED ^b^	18.6	20.4	24.9	
Some college or above	72.5	37.7	57.9	

* Values are presented as weighted mean ± standard error or weighted percent. ^a^ Poverty–income ratio (PIR): the ratio of income to the poverty threshold after accounting for inflation and family size [33]. PIR was divided into three levels: <1.00 (extremely poor, below the official poverty threshold), 1.0–1.84 (very and nearly poor), ≥1.85 (not poor). ^b^ GED: general equivalency diploma. BMI, body mass index.

**Table 2:** Estimated daily usual micronutrient intakes from foods among women aged 19–39 years by racial/ethnic groups, NHANES 2003–2014 (*N* = 3931).

Micronutrients	Mean ± SE ^a^			*p*-Value ^*^
	Non-Hispanic White (*n* = 1916)	Mexican-American (*n* = 916)	Non-Hispanic Black (*n* = 1099)	
Vitamin A, μg	557.8 ± 21.0	454.6 ± 16.7	504.1 ± 16.9	<0.0001
Vitamin B_2_, mg	2.0 ± 0.0	1.7 ± 0.0	1.8 ± 0.0	<0.0001
Vitamin B_6_, mg	1.7 ± 0.1	1.7 ± 0.1	1.7 ± 0.1	0.03
Vitamin B_12_, μg	4.4 ± 0.2	4.1 ± 0.2	4.3 ± 0.2	<0.0001
Vitamin C, mg	67.9 ± 3.2	89.4 ± 4.3	78.1 ± 3.4	<0.0001
Vitamin D, μg	3.9 ± 0.2	3.7 ± 0.2	3.8 ± 0.2	0.04
Folate, μg	367.7 ± 8.5	346.2 ± 9.8	356.6 ± 7.9	<0.0001
Calcium, mg	930.9 ± 23.0	849.2 ± 22.1	889.2 ± 20.5	<0.0001
Magnesium, mg	269.3 ± 5.6	262.8 ± 5.6	265.8 ± 4.8	0.02
Phosphorus, mg	1245.4 ± 24.0	1234.4 ± 27.6	1239.3 ± 23.4	0.26
Copper, μg	749.7 ± 32.7	720.4 ± 29.3	734.7 ± 30.0	0.12
Iron, mg	13.6 ± 0.3	13.8 ± 0.3	13.7 ± 0.2	0.34
Zinc, mg	10.3 ± 0.3	10.3 ± 0.3	10.3 ± 0.2	0.75
Energy, kcal	2053.0 ± 29.2	2099.4 ± 39.9	2075.9 ± 29.4	0.04

^a^ SE: standard error * *p*-value from linear regression by modeling estimated average usual dietary intake of micronutrient by racial/ethnic group.

**Table 3:** Estimated daily usual micronutrient intakes from foods, and micronutrient density among Mexican American women aged 19–39 years old by body weight status, NHANES 2003–2014 (*n* = 916).

Micronutrients	Mean ± SE ^a^			*p*-Value *
	Normal/Under-Weight (*n* = 291)	Overweight (*n* = 283)	Obese (*n* = 342)	
Vitamin A, μg	494.7 ± 40.7	467.9 ± 34.0	442.1 ± 31.7	0.0004
Vitamin B_2_, mg	1.8 ± 0.1	1.8 ± 0.1	1.7 ± 0.1	0.04
Vitamin B_6_, mg	1.7 ± 0.1	1.7 ± 0.1	1.7 ± 0.2	0.20
Vitamin B_12_, μg	3.9 ± 0.3	3.8 ± 0.3	3.7 ± 0.3	0.24
Vitamin C, mg	87.3 ± 8.6	79.3 ± 7.5	72.0 ± 8.2	0.0002
Vitamin D, μg	3.4 ± 0.3	3.2 ± 0.2	3.0 ± 0.3	0.03
Folate, μg	377.5 ± 19.5	372.3 ± 19.5	367.9 ± 22.7	0.24
Calcium, mg	907.6 ± 47.7	902.9 ± 45.9	895.9 ± 50.7	0.50
Magnesium, mg	264.1 ± 22.8	264.6 ± 9.4	264.9 ± 9.2	0.90
Phosphorus, mg	1228.5 ± 52.4	1248.6 ± 48.1	1269.3 ± 50.3	0.04
Copper, μg	1130.7 ± 124.7	1133.8 ± 112.8	1133.4 ± 115.1	0.06
Iron, mg	14.1 ± 0.7	14.0 ± 0.6	13.9 ± 0.7	0.58
Zinc, mg	9.9 ± 0.5	10.2 ± 0.5	10.4 ± 0.5	0.05
Energy, kcal	1947.3 ± 79.4	1979.1 ± 69.7	2007.5 ± 72.9	0.13

^a^ SE: standard error. * *p*-value from linear regression by modeling estimated average usual dietary intake of micronutrient by BMI status.

**Table 4:** Estimated daily usual micronutrient intakes from foods, and micronutrient density among non-Hispanic Black women aged 19–39 years by body weight status, NHANES 2003–2014 (*n* = 1099).

Micronutrients	Mean ± SE ^a^			*p*-Value *
	Normal/Under-Weight (*n* = 309)	Overweight (*n* = 250)	Obese (*n* = 540)	
Vitamin A, μg	419.7 ± 33.7	413.5 ± 27.4	408.3 ± 29.3	0.46
Vitamin B_2_, mg	1.5 ± 0.1	1.5 ± 0.1	1.5 ± 0.1	0.75
Vitamin B_6_, mg	1.6 ± 0.1	1.6 ± 0.1	1.6 ± 0.1	0.63
Vitamin B_12_, μg	3.9 ± 0.3	3.8 ± 0.2	3.7 ± 0.1	0.13
Vitamin C, mg	90.3 ± 9.4	88.3 ± 8.3	86.5 ± 8.5	0.34
Vitamin D, μg	3.1 ± 0.3	3.1 ± 0.2	3.0 ± 0.3	0.73
Folate, μg	318.1 ± 17.3	313.4 ± 13.3	307.8 ± 12.0	0.13
Calcium, mg	715.4 ± 41.1	731.0 ± 34.9	745.6 ± 36.5	0.14
Magnesium, mg	237.3 ± 9.9	239.6 ± 9.0	241.4 ± 10.0	0.42
Phosphorus, mg	1120.6 ± 44.5	1132.9 ± 36.6	1145.1 ± 42.1	0.37
Copper, μg	1060.5 ± 113.6	1079.8 ± 102.6	1094.8 ± 117.2	0.67
Iron, mg	13.4 ± 0.6	13.1 ± 0.4	12.8 ± 0.4	0.02
Zinc, mg	10.4 ± 0.5	10.1 ± 0.4	9.8 ± 0.4	0.01
Energy, kcal	2190.2 ± 87.6	2176.7 ± 71.0	2160.9 ± 70.3	0.43

^a^ SE: standard error. * *p*-value from linear regression by modeling estimated average usual dietary intake of micronutrient by BMI status.

## Supplementary Materials

The following are available online at https://www.mdpi.com/2072-6643/11/12/2846/s1, Figure S1: Percentage of non-Hispanic white women aged 19–39 years with dietary micronutrient intakes below EARs by body weight status, Body weight status categorized by BMI into three groups: normal/under-weight: BMI ≤ 25.0; overweight: 25.0 < BMI < 29.9; obese: BMI ≥ 30.0, Table S1: Estimated daily usual micronutrient intakes from foods, and micronutrient density among non-Hispanic White women aged 19–39 years by body weight status, NHANES 2003–2014 (*n* = 1916).

## References

[1] Christian P., Stewart C.P. (2010). Maternal micronutrient deficiency, fetal development, and the risk of chronic disease. J. Nutr..

[2] Cetin I., Berti C., Calabrese S. (2010). Role of micronutrients in the periconceptional period. Hum. Reprod. Update.

[3] Kontic-Vucinic O., Sulovic N., Radunovic N. (2006). Micronutrients in women’s reproductive health: Ii. Minerals and trace elements. Int. J. Fertil Womens Med..

[4] Zeisel S.H. (2009). Is maternal diet supplementation beneficial? Optimal development of infant depends on mother’s diet. Am. J. Clin. Nutr..

[5] Bartley K.A., Underwood B.A., Deckelbaum R.J. (2005). A life cycle micronutrient perspective for women’s health. Am. J. Clin. Nutr..

[6] Torheim L.E., Ferguson E.L., Penrose K., Arimond M. (2010). Women in resource-poor settings are at risk of inadequate intakes of multiple micronutrients. J. Nutr..

[7] Bastos Maia S., Rolland Souza A.S., Costa Caminha M.F., Lins da Silva S., Callou Cruz R.S.B.L., Carvalho Dos Santos C., Batista Filho M. (2019). Vitamin a and pregnancy: A narrative review. Nutrients.

[8] Jang W., Kim H., Lee B.E., Chang N. (2018). Maternal fruit and vegetable or vitamin c consumption during pregnancy is associated with fetal growth and infant growth up to 6 months: Results from the korean mothers and children’s environmental health (moceh) cohort study. Nutr. J..

[9] Liu C., Zhong C., Chen R., Zhou X., Wu J., Han J., Li X., Zhang Y., Gao Q., Xiao M. (2019). Higher dietary vitamin c intake is associated with a lower risk of gestational diabetes mellitus: A longitudinal cohort study. Clin. Nutr..

[10] Yin J., Dwyer T., Riley M., Cochrane J., Jones G. (2010). The association between maternal diet during pregnancy and bone mass of the children at age 16. Eur. J. Clin. Nutr..

[11] Kumanyika S.K., Obarzanek E., Stettler N., Bell R., Field A.E., Fortmann S.P., Franklin B.A., Gillman M.W., Lewis C.E., Poston W.C. (2008). Population-based prevention of obesity: The need for comprehensive promotion of healthful eating, physical activity, and energy balance: A scientific statement from american heart association council on epidemiology and prevention, interdisciplinary committee for prevention (formerly the expert panel on population and prevention science). Circulation.

[12] Pannu P.K., Calton E.K., Soares M.J. (2016). Calcium and vitamin d in obesity and related chronic disease. Adv. Food Nutr. Res..

[13] Hosseini B., Saedisomeolia A., Allman-Farinelli M. (2017). Association between antioxidant intake/status and obesity: A systematic review of observational studies. Biol. Trac. Elem. Res..

[14] Bento C., Mato A.C., Cordeiro A., Ramalho A. (2018). Vitamin a deficiency is associated with body mass index and body adiposity in women with recommended intake of vitamin a. Nutr. Hosp..

[15] Hales C.M., Carroll M.D., Fryar C.D., Ogden C.L. (2017). Prevalence of obesity among adults and youth: United states, 2015–2016. NCHS Data Brief.

[16] Ogden C.L., Carroll M.D., Fryar C.D., Flegal K.M. (2015). Prevalence of obesity among adults and youth: United states, 2011–2014. NCHS Data Brief.

[17] Rai D., Bird J.K., McBurney M.I., Chapman-Novakofski K.M. (2015). Nutritional status as assessed by nutrient intakes and biomarkers among women of childbearing age--is the burden of nutrient inadequacies growing in america?. Public Health Nutr..

[18] Storey M.L., Anderson P.A. (2016). Vegetable consumption and selected nutrient intakes of women of childbearing age. J. Nutr. Educ. Behav..

[19] Yang Q.H., Carter H.K., Mulinare J., Berry R.J., Friedman J.M., Erickson J.D. (2007). Race-ethnicity differences in folic acid intake in women of childbearing age in the united states after folic acid fortification: Findings from the national health and nutrition examination survey, 2001–2002. Am. J. Clin. Nutr..

[20] Groth S.W., Stewart P.A., Ossip D.J., Block R.C., Wixom N., Fernandez I.D. (2017). Micronutrient intake is inadequate for a sample of pregnant african-american women. J. Acad. Nutr. Diet.

[21] U.S. Department of Health Human Services, U.S. Department of Agriculture (2015). 2015–2020 Dietary Guidelines for American.

[22] Johnson C.L., Dohrmann S.M., Burt V., Mohadjer L.K. (2014). National health and nutrition examination survey: Sample design, 2011–2014. Vital Health Stat 2.

[23] Centers for Disease Control Prevention (2014). National Health and Nutrition Examination Survey (nhanes): Anthropometry Procedures Manual.

[24] James P.T., Leach R., Kalamara E., Shayeghi M. (2001). The worldwide obesity epidemic. Obes. Res..

[25] Ahluwalia N., Dwyer J., Terry A., Moshfegh A., Johnson C. (2016). Update on nhanes dietary data: Focus on collection, release, analytical considerations, and uses to inform public policy. Adv. Nutr..

[26] U.S. Department of Agriculture, Agricultural Research Service Usda National Nutrient Database for Standard Reference. https://www.ars.usda.gov/northeast-area/beltsville-md-bhnrc/beltsville-human-nutrition-research-center/nutrient-data-laboratory/docs/usda-national-nutrient-database-for-standard-reference/.

[27] Institute of Medicine (2006). Dietary Reference Intakes: The Essential Guide to Nutrient Requirements.

[28] Tooze J.A., Midthune D., Dodd K.W., Freedman L.S., Krebs-Smith S.M., Subar A.F., Guenther P.M., Carroll R.J., Kipnis V. (2006). A new statistical method for estimating the usual intake of episodically consumed foods with application to their distribution. J. Am. Diet. Assoc..

[29] Tooze J.A., Kipnis V., Buchman D.W., Carroll R.J., Freedman L.S., Guenther P.M., Krebs-Smith S.M., Subar A.F., Dodd K.W. (2010). A mixed-effects model approach for estimating the distribution of usual intake of nutrients: The NCI method. Stat. Med..

[30] Herrick K.A., Rossen L.M., Parsons R., Dodd K.W. (2018). Estimating usual dietary intake from national health and nutrition examination survey data using the national cancer institute method. Vital Health Stat..

[31] Subcommittee on Criteria for Dietary Evaluation, Coordinating Committee on Evaluation of Food Consumption Surveys, Food and Nutrition Board, Commission on Life Science, National Research Council (1986). Nutrient Adequacy: Assessment Using Food Comsumption Surveys.

[32] SAS Institute Inc. (2015). Base SAS 9.4 Procedures Guide.

[33] U.S. Census Bureau Ratio of Income to Poverty Level. http://www.census.gov/hhes/income/defs/ratio.html.

[34] Tidwell D.K., Valliant M.W. (2011). Higher amounts of body fat are associated with inadequate intakes of calcium and vitamin d in african american women. Nutr. Res..

[35] Zemel M.B., Richards J., Milstead A., Campbell P. (2005). Effects of calcium and dairy on body composition and weight loss in african-american adults. Obes. Res..

[36] García O.P., Ronquillo D., Caamaño Mdel C., Camacho M., Long K.Z., Rosado J.L. (2012). Zinc, vitamin a, and vitamin c status are associated with leptin concentrations and obesity in mexican women: Results from a cross-sectional study. Nutr. Metab..

[37] Chambers E.C., Heshka S., Gallagher D., Wang J., Pi-Sunyer F.X., Pierson R.N. (2006). Serum iron and body fat distribution in a multiethnic cohort of adults living in new york city. J. Am. Diet. Assoc..

[38] Institute of Medicine (US) Panel on Micronutrients (2001). Dietary Reference Intakes for Vitamin A, Vitamin K, Arsenic, Boron, Chromium, Copper, Iodine, Iron, Manganese, Molybdenum, Nickel, Silicon, Vanadium, and Zinc.

[39] Ross A.C., Caballero B., Cousins R.J., Tucker K.L., Ziegler T.R. (2014). Modern Nutrition in Health and Disease.

[40] Pang X.Y., Wang S., Jurczak M.J., Shulman G.I., Moise A.R. (2017). Retinol saturase modulates lipid metabolism and the production of reactive oxygen species. Arch Biochem. Biophys..

[41] Arab L., Carriquiry A., Steck-Scott S., Gaudet M.M. (2003). Ethnic differences in the nutrient intake adequacy of premenopausal us women: Results from the third national health examination survey. J. Am. Diet. Assoc..

[42] Cha S., Kang J.H., Lee J.H., Kim J., Kim H., Yang Y.J., Park W.Y., Kim J. (2018). Impact of genetic variants on the individual potential for body fat loss. Nutrients.

[43] Oh R., Brown D.L. (2003). Vitamin b12 deficiency. Am. Fam. Physician.

[44] Crider K.S., Bailey L.B., Berry R.J. (2011). Folic acid food fortification-its history, effect, concerns, and future directions. Nutrients.

[45] Dietrich M., Brown C.J., Block G. (2005). The effect of folate fortification of cereal-grain products on blood folate status, dietary folate intake, and dietary folate sources among adult non-supplement users in the united states. J. Am. Coll. Nutr..

[46] Tinker S.C., Cogswell M.E., Devine O., Berry R.J. (2010). Folic acid intake among U.S. Women aged 15–44 years, national health and nutrition examination survey, 2003–2006. Am. J. Prev. Med..

[47] Murakami K., Livingstone M.B. (2015). Prevalence and characteristics of misreporting of energy intake in us adults: Nhanes 2003–2012. Br. J. Nutr..

[48] Roark R.A., Niederhauser V.P. (2013). Fruit and vegetable intake: Issues with definition and measurement. Public Health Nutr..

