# Supplementary material for: Comparison of Dietary Micronutrient Intakes by Body Weight Status among Mexican-American and Non-Hispanic Black Women Aged 19–39 Years: An Analysis of NHANES 2003–2014
Source: Nutrients. 2019 Nov 20;11(12):2846. doi: 10.3390/nu11122846 (PMC6950012; doi:10.3390/nu11122846)
Supplement: Supplementary file 1 [file nutrients-11-02846-s001.pdf]

Table S1. Estimated daily usual micronutrient intakes from foods, and micronutrient density among non-Hispanic White women aged 19-39 years by body weight status, NHANES 2003-2014 (n = 1,916)

Micronutrients	Mean ± SE [a]			*p*-value[*]
	Normal/under-weight (n = 908)	Overweight (n = 441)	Obese (n = 567)	
Vitamin A, μg	600.4 ± 31.0	572.9 ± 25.8	547.1 ± 27.7	0.0002
Vitamin B_2, mg	2.1 ± 0.1	2.0 ± 0.1	2.0 ± 0.1	0.049
Vitamin B_6, mg	1.8 ± 0.1	1.7 ± 0.1	1.7 ± 0.1	0.0004
Vitamin B_{12}, μg	4.6 ± 0.2	4.6 ± 0.2	4.6 ± 0.2	0.81
Vitamin C, mg	71.4 ± 4.2	66.2 ± 3.8	61.3 ± 4.4	<0.0001
Vitamin D, μg	4.2 ± 0.3	4.1 ± 0.2	4.1 ± 0.3	0.44
Folate, μg	389.9 ± 11.5	369.1 ± 10.9	349.3 ± 12.2	<0.0001
Calcium, mg	967.6 ± 34.4	957.4 ± 29.8	948.7 ± 33.3	0.21
Magnesium, mg	282.5 ± 8.3	272.5 ± 6.6	262.6 ± 7.0	<0.0001
Phosphorus, mg	1267.3 ± 35.6	1266.7 ± 30.9	1266.2 ± 33.8	0.92
Copper, μg	793.3 ± 33.4	756.9 ± 29.0	723.0 ± 28.6	0.002
Iron, mg	13.9 ± 0.4	13.6 ± 0.3	13.3 ± 0.4	0.001
Zinc, mg	10.3 ± 0.3	10.4 ± 0.4	10.4 ± 0.4	0.53
Energy, kcal	2070.1 ± 43.1	2047.7 ± 35.3	2025.7 ± 38.9	0.08

[a] SE: standard error

[**] *p*-value from linear regression by modeling estimated average usual dietary intake of micronutrient by BMI status

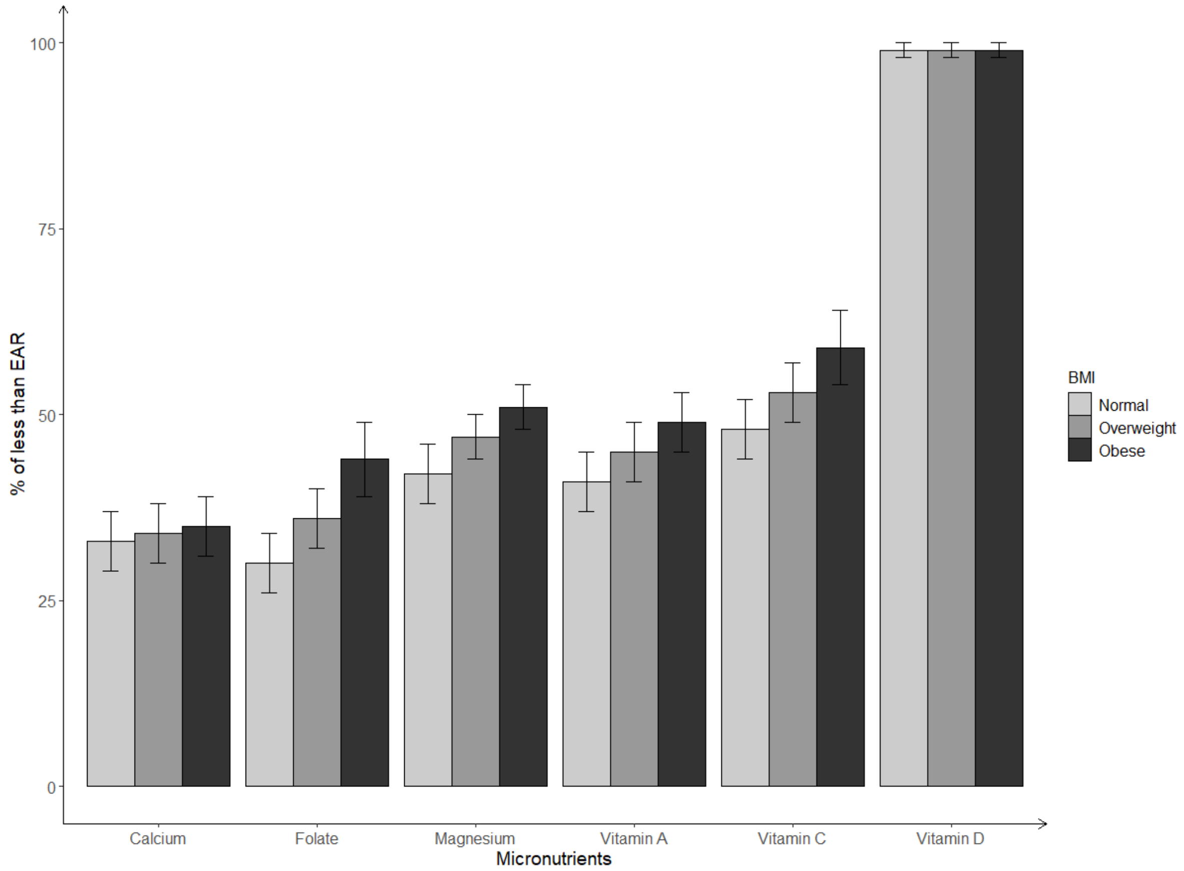

Figure S1. Percentage of non-Hispanic white women aged 19-39 years with dietary micronutrient intakes below EARs by body weight status [a].

[a] Body weight status categorized by BMI into three groups: normal/under-weight: BMI ≤25.0; overweight: 25.0<BMI<29.9; obese: BMI≥30.0.
